# Can Interacting with Animals Improve Executive Functions? A Systematic Review

**DOI:** 10.3390/ani13132080

**Published:** 2023-06-23

**Authors:** Deanna Tepper, Joanna Shnookal, Tiffani Howell, Pauleen Bennett

**Affiliations:** School of Psychology and Public Health, La Trobe University, Bendigo, VIC 3550, Australia; j.shnookal@latrobe.edu.au (J.S.); t.howell@latrobe.edu.au (T.H.); pauleen.bennett@latrobe.edu.au (P.B.)

**Keywords:** cognition, development, human–animal relationship, human–pet relationship, animal-assisted services, children, adolescence, older adults

## Abstract

**Simple Summary:**

Executive functions are cognitive processing skills associated with planning, problem-solving, decision making, and regulating behaviour. For some individuals these abilities may be impaired, which can have negative long-term impacts. It has been proposed that interacting with animals may provide an opportunity to strengthen these skills. A systematic review was carried out to assess the ways in which interacting with animals may improve executive functions. This review included 23 studies exploring executive functions across three contexts: the human–pet relationship, the presence of an animal, and involvement in an animal-assisted service. There is some evidence to suggest that interacting with an animal may be beneficial for older adults, whilst horseback riding seems particularly beneficial for children; however, the overall methodological rigour is limited.

**Abstract:**

There has been growing interest in the potential benefits of using human–animal interactions to improve executive functions: cognitive processes that allow individuals to plan, solve problems, and self-regulate behaviour. To date, no comprehensive review has been conducted. The purpose of this study was to evaluate existing literature, adopting broad inclusion criteria. Following Preferred Reporting Items for Systematic Reviews and Meta-Analyses (PRISMA) guidelines, 16 papers were identified from peer-reviewed literature. Additional papers were identified from grey literature, including 6 dissertations and 1 thesis. A review of these 23 studies found that human–animal interactions and executive functions are investigated in three main ways: (1) exploring the potential benefits of the human–pet relationship, (2) exploring the impact of an animal’s presence during administration of executive function tests, and (3) evaluating the efficacy of animal-assisted services (e.g., animal-assisted therapy) on executive functions. Five of the included studies reported a significant improvement across all measured domains of executive functions, but effect sizes were underreported. Comparatively, 9 studies reported mixed findings, *d* = 0.32–0.55, while 8 studies reported no significant results. The overall rigour of the research was limited, with great heterogeneity between the study methodologies and outcome measures used. It is recommended that future studies utilise high-quality research methodologies through the use of randomisation, pre- and postmeasures, and appropriate control conditions, where possible.

## 1. Introduction

‘Executive functions’ (EFs) is an umbrella term for a set of cognitive processes that allow individuals to regulate their behaviour, plan, solve problems, and make decisions [1,2]. While the conceptualisation of EFs is subject to ongoing debate [3], many developmental and cognitive researchers have defined EFs as comprising three core processes: (1) working memory (WM), the ability to monitor new information and revise old information; (2) inhibition, the ability to inhibit prepotent responses; and (3) cognitive flexibility (also known as set shifting), being able to move between tasks and adapt behaviour [4,5,6].

Typically, EFs begin to develop during early childhood and continue developing into early adulthood [7,8,9,10]. Whilst EFs remain relatively stable throughout adulthood, studies have reported a mild age-related decline in older adults [11,12]. Impairments in EFs, usually associated with damage to the prefrontal cortex of the brain, can, however, occur at any age. These impairments can potentially have a significant impact on academic and workplace success, quality of life, and adaptive functioning [1,13,14]. Additionally, impairments in EFs have been associated with dementia [15], mood and anxiety disorders [16,17], and post-traumatic stress disorder (PTSD) [18] as well as with neurodevelopmental conditions such as attention-deficit/hyperactivity disorder (ADHD) [19] and autism spectrum disorder (ASD) [20]. Fortunately, research suggests that EFs can be improved with a variety of interventions, including through computerised training programs and targeted classroom curricula; for a review, see [21]. Unfortunately, many of these programs are difficult to access, and the long-term benefits of such programs are still unknown [21].

In the last decade, both caregiving for a pet [22] and the incorporation of animals into therapeutic and educational settings [23] have become more common. Extensive research has explored the purported benefits of interacting with animals, with reviews suggesting that interacting with animals can improve behavioural symptoms associated with dementia and Alzheimer’s disease [24,25] and ADHD and ASD [26,27,28,29,30]. Such reviews have also explored the cognitive benefits of interacting with animals, with some research finding improvements in attention, language, visuospatial ability, and overall memory [25]. It is also possible that interacting with animals can be used to improve EFs, particularly as such conditions, as previously stated, have been associated with impairments or delays in EFs. As many individuals reside with a pet [22] or have other forms of access to an animal [23], human–animal interactions may be an accessible and relatively low-cost way to improve EFs.

Several hypotheses have been proposed to explain why this is theoretically possible. First, better physical and mental health is associated with stronger EF ability [1]. It has previously been suggested that activities that are enjoyable and engaging and encourage social interaction can improve EFs, with some evidence to support the use of martial arts and yoga programs [21]. Relevant to this, interacting with animals has been shown to improve mood [31,32], decrease stress [31,33], reduce feelings of loneliness and isolation [34,35], and, in some circumstances, improve physical well-being [36]. As such, it is possible that these benefits of human–animal interactions may positively impact EFs.

Second, safely interacting with any animal requires individuals to monitor and adjust their own behaviour in response to the animal and to utilise self-control [37]. In turn, this may improve inhibition and cognitive flexibility skills. Third, it has been suggested that activities related to pet care may improve overall cognitive skills [38,39]. For example, feeding a pet requires an individual to remember the feeding schedule, retrieve and pour the correct amount of food into a bowl, provide the food to the pet, and clean up afterward. Practicing such skills may result in generalised improvements in EFs.

As discussed, many reviews have explored the benefits of human–animal interactions across a range of contexts. This includes broader reviews on ASD [26,27,28], ADHD [29], and dementia symptomatology [24] as well as more specific reviews exploring the benefits of human–animal interactions in classroom settings [40] and on reading ability [41]. To date, however, no review has explored how human–animal interaction might impact EFs. This is important to explore, as EF ability is associated with all these conditions and outcomes [42,43,44]. The first aim of this systematic review was therefore to identify and summarise the current literature available on human–animal interactions and EFs. Furthermore, the secondary aim of this review was to critically evaluate the efficacy of using interaction with animals to improve EFs, assessing the context, setting and populations for whom such interactions or programs may most benefit.

## 2. Materials and Methods

A literature search was conducted using the Preferred Reporting Items for Systematic Reviews and Meta-Analyses (PRISMA) guidelines [45]. Relevant literature was first identified through electronic searches of databases, including Google Scholar, PubMed, Web of Science, Science Direct, and PsycINFO from inception to September 2022. The HABRI Central (habricentral.org/resources) and WALTHAM (waltham.com/resources) websites, which compile research on human–animal relationships, were also searched. The search terms used to collate the literature were ‘animal-assisted intervention’, ‘animal-assisted therapy’, ‘pet’, ‘companion animal’, and ‘human–animal interaction’, in combination with ‘executive function *’, ‘working memory’, ‘inhibit *’, ‘cognitive flexibility’, and ‘attention’.

The following criteria were used to select relevant papers for review: (1) publication in English; (2) collection of empirical, quantitative data on overall EF ability, a specific EF process (e.g., working memory), or the prefrontal cortex with explicit reference to EFs; and (3) reference to human–animal interactions (HAI) or involvement in an animal-assisted service (AAS), such as animal-assisted therapy, animal-assisted education, and animal-assisted activities. The participant demographic was kept broad across age, ability, and potential diagnosis, and different types of human–animal interactions (e.g., involvement in a therapeutic program or interaction with a pet) were explored. Nonpublished works were retained to avoid publication bias.

Studies were excluded if they did not include at least one adequate measurable assessment of executive function. For example, the Mini-Mental State Examination (MMSE) [46] is a screening tool for cognitive impairment in older adults and contains an item on working memory. However, the MMSE also assesses other cognitive domains and presents cognitive ability as a total score. As such, studies using the MMSE or other similar tools cannot be used to determine any impact on EF ability alone. Similarly, studies that focused on overall ASD, ADHD, or dementia symptoms were excluded if they did not specifically focus on EF.

The quality of the included studies was assessed, where possible, using various checklists designed by the Joanna Briggs Institute (JBI), including the Critical Appraisal Tool for Assessment of Risk of Bias for Randomised Controlled Trials [47], the Checklist for Quasi-Experimental Studies [48], and the Checklist for Case Reports [49]. By asking 8 to 13 closed-ended questions, the JBI checklists evaluate the quality of the study design, the quality of comparison groups, and the reliability of the outcome measures and statistical analyses. Appropriate JBI checklists were not available for all research designs included in this review, particularly studies in which the effects of the human–pet relationship were evaluated.

## 3. Results

### 3.1. Study Selection

**Figure 1:**
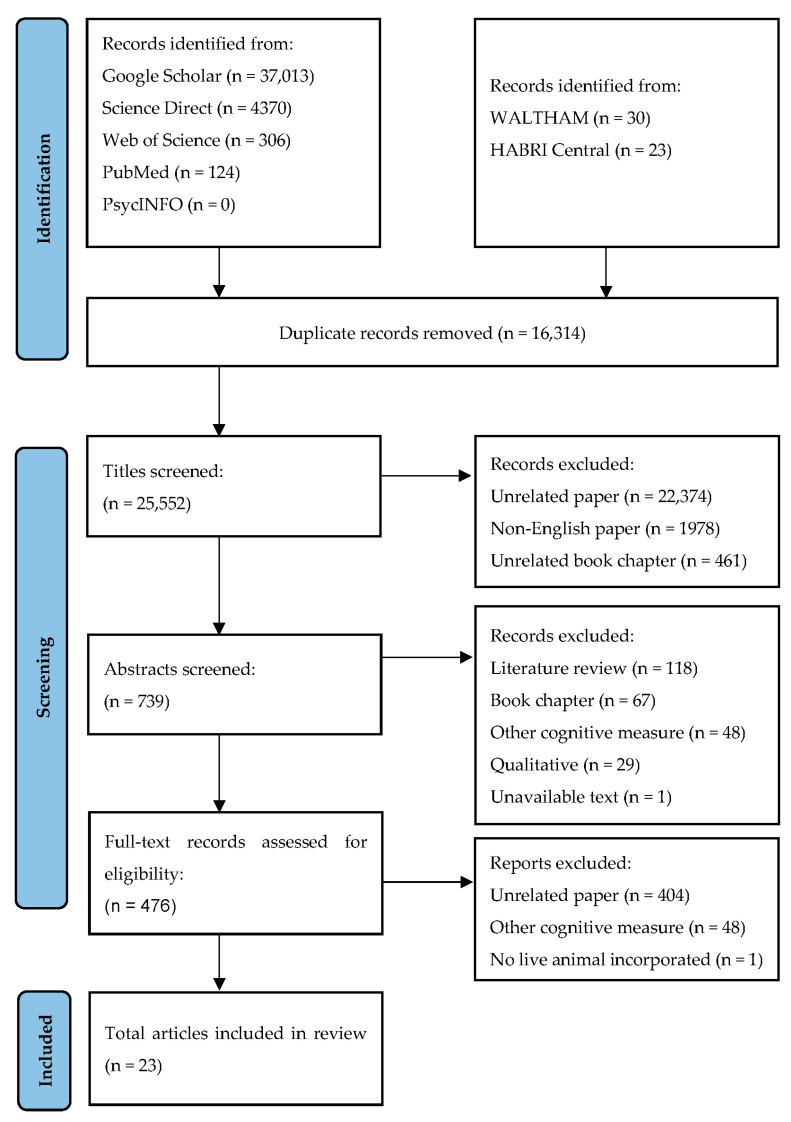
Article-selection process detailing the number of articles included and excluded at each step of the review using the PRISMA flow guidelines. ‘Other cognitive measure’ refers to the use of the measures such as the Mini-Mental State Examination, which contains an item measuring executive functions but provides a total cognitive score.

The initial search identified 41,866 articles, with Figure 1 showing the study selection process. Due to the inclusion of the search term ‘attention’, a large number of papers were collated on ADHD literature as well as literature exploring attention and regulatory behaviours in educational settings. This is not uncommon in EF literature, with other reviews reporting large initial searches [50,51]. As per our exclusion criteria, papers were excluded if they did not specifically focus on EFs.

The initial searching and screening were performed by the first author (DT) following a three-stage approach (title, abstract, full text), during which the sample was reduced to 476 potentially relevant articles. This sample included book chapters and literature reviews that were screened for additional records before being considered for inclusion; none were identified. Following the approach of other reviews with a larger search size, a randomised subset of the full-text papers (*n* = 73; 15%) were independently screened by the second author (JS). At the conclusion of the screening, a total of 16 peer-reviewed journal articles were retained, along with 6 dissertations and 1 thesis. The final sample size was therefore 23 works, published between 2014 and 2022. Of these studies, 16 (69.6%) were published after 2018. At time of publication, the corresponding authors were located in the United States of America (*n* = 8, 34.8%), the United Kingdom (*n* = 4, 17.4%), Israel (*n* = 3, 13.0%), Australia (*n* = 3, 13.0%), and Belgium, Italy, Japan, South Korea, and Switzerland (*n* = 1 each, 4.3%).

### 3.2. Study Characteristics

A review of all included studies (*N* = 23) found that researchers explored EFs in three main ways: (1) examining associations between the human–pet relationship and EFs, (2) testing whether EF task performance is improved in the presence of an animal, and (3) examining EFs following involvement in an animal-assisted service. Due to these different research questions and therefore the differences in study design and findings, a direct comparison of these studies is difficult. It was also impossible to conduct a meta-analysis. Instead, the three types of research questions are discussed separately in the following analysis. Finally, 11 of the included papers (47.8%) did not provide effect sizes; where possible, when adequate data was presented, effect sizes were manually calculated [52,53].

### 3.3. Research Question 1: The Human–Pet Relationship

Four studies examined the human–pet relationship. Two studies examined the human–pet relationship in older adults, with sample sizes ranging from 52 to 88 participants and with approximately half of the participants from each study caregiving a pet. Participants were recruited from individuals receiving support for physical and/or mobility limitations [39] and stroke rehabilitation [54]. Two studies examined the human–pet relationship in children, with one of these testing whether children who engage in a greater number of household chores have stronger EF skills, with household chores including pet care–related activities (e.g., feeding a pet, taking a pet for a walk) [38]. The final study examined longitudinal data from the Avon Longitudinal Study of Parents and Children (ALSPAC) birth cohort study [55]. While the ALSPAC study initially recruited 14,541 pregnant women, only 13,557 participants provided initial data on whether they kept a pet. Of these participants, 58% reported keeping a pet during gestation. By the time the child was age 10, 74% of participants had a pet. This remained relatively stable, with 72% of the now adolescents (52% male) self-reporting living with a pet between the ages of 11 and 18 years [55].

The results suggest that the human–pet relationship may benefit older adults, with both Branson et al. [39] and Demeter [54] finding that older adults with a pet had stronger EF ability across executive control and sustained attention than older adults without a pet. In comparison, it does not appear that caring for a pet influences children’s EFs. In the study by Tepper and colleagues [38], there was no evidence to suggest that engagement in pet care chores predicted EFs, whereas engagement in other household chore types (e.g., children making their own beds) was a predictor of EF ability. Supporting this, the longitudinal study by Purewal [55] found no significant developmental differences between individuals with a pet, regardless of the pet species, versus individuals without a pet. The results for the four studies exploring the human–pet relationship are shown in Table 1.

The quality of the four human–pet relationship studies could not be assessed using JBI checklists, which are not designed for longitudinal studies or cross-sectional research not pertaining to epidemiology and disease prevalence [49,58]. For these studies, we instead note the limitations inherent in these studies, including the use of convenience sampling [39,54] and that the cross-sectional data means that casual interpretations cannot be made [38,39,54,55].

### 3.4. Research Question 2: Presence of an Animal

Several studies (*n* = 6) examined whether interacting with an animal impacts performance on an EF task. These studies were further divided into two categories: completing EF tasks whilst in the presence of an animal (*n* = 4, 66.7%) [59,60,61,62] and completing EF tasks immediately after interacting with an animal (*n* = 2, 33.3%) [63,64]. Two of the studies did not provide enough detailed information to determine the time spent in the overall experiment (e.g., greeting researchers, receiving instructions, testing with animal present, finishing experiment) versus actual time spent directly interacting with the animal. Of the four studies that provided information about the total time spent with the animal [59,62,63,64], interactions ranged between 3 min and 15 min in length (*M*_time_ = 6.25, *SD* = 5.85).

For the three studies examining children, the mean age was 10.18 years (*SD* = 2.33), with two of these studies recruiting from populations with a diagnosis of neurodevelopmental and/or behavioural disorders [59] and a learning disability [61]. There appears to be some evidence that the presence of an animal can improve cognitive flexibility and inhibitory skills [59] and WM [61,63] for children. In addition, Hediger and Turner [63] found that brain activity, as measured by neurofeedback in the frontal lobe and the prefrontal cortex, the area of the brain associated with EFs, was greater when interacting with a real dog versus a robotic control.

For the studies exploring EFs in adults, all participants were recruited from university students (*M*_age_ = 20.09, *SD* = 1.00). In a neuroimaging study by Nagasawa et al. [62], participants demonstrated greater prefrontal cortex activation when interacting with a cat, with the authors concluding that this may transfer to overall EF ability. In comparison, Thayer and Stevens [64] found no significant changes in EFs when interacting with a dog across two independent experiments, as measured by performance on WM tasks. Similarly, results from Gee et al.’s [60] counterbalanced study were mixed, with participants improving in EF task performance when either the human collaborator or the dog were simply sitting next to the participant. However, performance was poorest when participants were required to maintain physical contact with the dog compared to when they were maintaining physical contact with the human collaborator [60]. The study characteristics and results of all six studies are presented in Table 2.

As shown in Table 3, the overall quality of assessed studies was moderate to high, with all studies measuring outcomes and conducting analyses appropriately. However, for the four studies using a randomised controlled trial design, all were ambiguous in terms of how participants were randomly assigned to conditions. Due to the nature of the studies and the research question, blinding the participants to the presence of the animal was not possible; one study, however, did attempt to obfuscate the aim of the study by telling the participants that the experimenter needed to bring their dog to the testing session, as the dog could not be left alone at home [63]. Additionally, outcome assessors were not blinded to the conditions. All but one study used validated measures. In Gee et al.’s study [60], participants played a digital version of the classic electronic Simon game, wherein participants reproduce, in order, a pattern of lights presented on a four-quadrant touchpad; the Simon game has been well represented in past research, but this digital version has not been validated [65,66]. The two quasi-experimental studies, as seen in Table 3, were also moderate in quality, lacking the inclusion of appropriate control conditions.

### 3.5. Research Question 3: Animal-Assisted Service

Thirteen studies examined the efficacy of animal-assisted services on EFs. Most of these studies examined the use of animal-assisted services with children (*n* = 10, 76.9%; *M*_age_ = 9.51, SD = 9.04), while two (15.4%) studies used adult participants; of these two papers, one recruited from younger adults [67], while another paper recruited older adults [68]. Finally, one study examined the impact of an animal-assisted service on both adult and child participants [69]. Among all the studies, the sample size ranged from 2 to 309 participants (*M* = 66.85, SD = 86.43). For the 12 studies that provided information on the total program duration, the programs ranged from 4 to 52 weeks in length, with an average of 13.5 weeks (SD = 13.76). Of the 10 studies that reported the frequency of sessions, most ran only one day per week (*n* = 8, 80%; *M* = 1.20, *SD* = 0.42). The sessions ranged from 20 to 120 min in length (*M* = 51.50, *SD* = 30.20).

Most of the reviewed studies involved horses and took place at a riding centre (*n* = 8, 61.5%). Excluding Norwood et al. [70] and Schroeder [69], 6 of the studies were designed to address specific therapeutic goals, such as improving social and communication skills, and self-regulation skills. Four studies (30.8%) incorporated a dog, with settings including schools (*n* = 2, 50.0%), a university campus (*n* = 1, 25.0%), and a clinic (*n* = 1, 25.0%). The number of dogs involved in these studies ranged from 2 to 27. Typically, the dogs were recruited from therapy dog organisations and accompanied by a trained handler. However, in 1 study the dogs were recruited from a nonprofit rescue organisation, with interactions overseen by a veterinarian [71]. Across all studies, interactions with the dogs varied. Finally, 1 study explored the benefits of raising 4–5 garden crickets over an 8-week period [68]. A research assistant ensured compliancy with the program through weekly telephone counselling [68]. The study characteristics and findings for all 13 studies are presented in Table 4.

The overall quality of the assessed studies was moderate to high. Overall, strengths of the studies included the use of reliable and validated measures of EFs and the use of appropriate statistical analyses. For the randomised controlled trials, there was once again some ambiguity over how participants were allocated to conditions, with only one study specifying that a computerised, random number generator had been used [68]. Due to the nature of the programs, blinding the participants and those delivering the programs to the participants was not feasible, although in one study the authors informed all participants that they would have the opportunity to interact with animals but blinded the participants as to the timing and amount of human–dog interaction they would receive [67]. This was done in an attempt to prevent condition-specific attrition [67]. Finally, related to blinding, it was often unclear whether the outcome assessors were aware of which conditions the participants had been assigned.

The quasi-experimental research and case studies were of moderate quality. A common limitation was the absence of a control condition. Norwood et al. [70] attempted to address this by testing a smaller subset of the participants six weeks prior to the program commencing, allowing participants to act as their own control across three time points (T0-T1-T2). The study by Schroeder [69] was the only study to include a traditional control condition; however, some of the participants in the experimental condition were already participating in horseback riding. Finally, the 2 case studies would have benefitted from more information about the participants [72] and the horseback riding program [73], respectively. Table 5 presents the quality assessments for the 13 studies examining the impact of animal-assisted services.

**Table 4:** Study characteristics, methodology, and key findings for *n* = 13 studies examining the impact of an animal-assisted service.

First Author (Year)	Participants				Animal-Assisted Service Characteristics	Methodology	Outcome Measure	Outcomes
	*N*	Age in Years (*M ± SD*)	Gender (% Male)	Diagnosis or Risk				
Aviv (2021) [74]	123	8.95 ± 1.68	72.36	ADHD	Therapeutic horseback riding, 1 × 30-min session per week over 20 weeks. No information on number of horses involved.	Random assignment to one of two conditions: equine-assisted therapy plus medication as usual; medication as usual control.	BRIEF (Hebrew version)	Significant time × group difference across all BRIEF subtests *. No effect size reported.
Borgi (2016) [75]	28	8.60 ± 1.70	100.00	ASD	Therapeutic horseback riding, 1 × 60–70-min session per week over 25 weeks. A total of 20 horses involved in study.	Random assignment to one of two conditions: equine-assisted therapy; waitlist control.	Tower of London task	Significant time × group difference in planning time *. No effect size reported. No significant differences in execution time, total time taken, number of correct solutions, number of rule violations, total number of moves.
Dimolareva (2020) ^a^ [76]	157	9.12 ± 0.91	81.50	Neurodevelopmental and behavioural disorders ^b^	Interaction with therapy dogs, including playing games or teaching the dogs new tricks, 2 × 20-min session per week over 4 weeks. No information on number of dogs involved.	Random assignment to one of three conditions, following stratification according to ability, socioeconomic status, and caring for a dog: dog intervention; relaxation intervention; no treatment control.	Fruit Stroop task	No significant differences in processing speed or task interference score.
Gilboa (2020) [77]	25	9.44 ± 1.75	88.00	ADHD	Therapeutic horseback riding, 1 × 45-min session per week over 12 weeks. No information on number of horses involved.	Pre- and postintervention without control group.	BRIEF (Hebrew version)	Significant improvement in initiation *, WM *, monitoring *, metacognitive index **, global executive composite *. Cohen’s *d* = 0.32–0.49 (small to medium effect). No significant differences in shifting, inhibition, emotional control, planning/organising.
Koenigseder (2016) ^a^ [72]	2	Not reported (7–9 years)	50.00	Developmental delays, learning difficulties	Therapeutic horseback riding, 1 × 60-min session per week over 6 weeks. No information on number of horses involved.	Pre- and postintervention, using case study (*N* = 2) design.	BRIEF	Minor improvements in EF domains reported, but no *p*-values or effect sizes included by original authors. Subsequent analyses by the authors of this review revealed significant improvement across time for one participant **.
Naste (2018) [73]	2	Not reported (11–12 years)	0.00	PTSD, learning difficulties	Session data not reported, total program 9–12 months. No information on number of horses involved.	Pre- and postintervention, using longitudinal case study (*N* = 2) design.	BRIEF-P	Mixed findings across EF domains reported, but no *p*-values or effect sizes included by original authors. Not enough data provided for subsequent analysis.
Norwood (2021) [70]	50	13.88 ± Not reported	58.00	At-risk children ^c^	Horseback riding program with no therapeutic element, 1 × 120-min session per week over 7 weeks. No information on number of horses involved.	Pre- and postprogram. Data was collected from some participants (*n* = 9, 18%) 6 weeks prior to starting the program to allow them to act as their own control.	BRIEF	Significant improvement across 10 EF domains * except for emotional control. Cohen’s *d* = −0.33–−0.56 (small to medium effect).
Panczykowski (2021) [78]	9	11.22 ± 1.92	77.80	Neurodevelopmental disorders, intellectual disabilities ^d^	Therapeutic horseback vaulting intervention, 1 × 60-min session per week over 10 weeks. No information on number of horses involved.	Quasi-experimental pre-and postdesign, with participants recruited from individuals who already participated in horseback riding. No control group.	BRIEF-2	Parents reported no significant differences across all EF domains. Horseback riding instructors reported improvements in self-monitoring *, shifting *, emotional control *, initiation *, WM *, planning *, and organization of materials *. No effect sizes reported by original authors; effect sizes subsequently calculated by authors of this review as Cohen’s *d* = 1.16–2.94 (large). Small sample size noted.
Park (2019) [68]	36	Not reported (>60 years)	0.00	None	Home-based AAS. Participants raised 4–5 oriental garden crickets over an 8-week period.	Random assignment to one of two conditions: insect-rearing condition; meditative music control group.	fMRI neuroimaging; WCST	Significant task improvement in participants with poorer baseline **. No effect size reported. Increased prefrontal cortex activation for participants with poorer baseline *.
Pendry (2021) [67]	309	19.00 ± Not reported	19.42	At-risk students	Meet-and-greet with the dog-handler team and opportunities to pet dogs whilst working through mindfulness activities, 1 × 60-min session per week over 4 weeks. Seven handler-dog teams involved per session.	Random assignment to one of three conditions: dog intervention (HAI-only); dog intervention + Academic Stress Management program (HAI-Enhanced); Academic Stress Management (ASM) program control condition.	BRIEF-A	Significant improvement in global EF * and metacognition * for at-risk participants in HAI-only experimental condition, Cohen’s *d* = 0.53 and 0.52 (medium effect). Improvements in global EF * and metacognition ** for at-risk participants in HAI-only experimental condition maintained at 12-week follow-up, Cohen’s *d* = 0.47 and 0.55 (medium effect).
Schroeder (2015) ^a,e^ [69]	Child: 56 Adult: 109	Child: 9.50 ± 1.80 Adult: 18.83 ± 1.32	Child: 50.00 Adult: 36.00	None	Horseback riding program. Minimal information reported.	Quasi-experimental design, with participants recruited from individuals who did or did not already participate in horseback riding. Included both child and adult participants.	BRIEF; BRIEF-A; Digit and letter span; ANT; ANT-C	Child participants: Significant group difference in WM digit span task for children *, η^2^ = 0.31 (large effect). No significant improvements in EF skills as reported by parents. Adult participants: No group difference in WM digit span task. Participants in experimental condition self-reported poorer organisational skills **, η^2^ = 0.07 (medium effect).
Tepper (2021) [79]	63	7.43 ± 0.62	42.90	None	Interaction with therapy dogs, including teaching the dogs tricks and reading to the dogs, 2 × 20-min sessions per week over 4 weeks. Seven dogs involved.	Random assignment to one of three conditions, following matching according to age and gender: dog training; reading to dog; dogs present while completing class-as-usual control.	WISC-IV digit span; TEA-Ch opposite worlds	Significant improvement across time for all conditions, partial η^2^ = 0.01–0.75 (small to large). Significant improvements in inhibition *** and cognitive flexibility *** and WM ** for participants with poorer baseline in dog training condition, Pearson’s *r* = −0.54–−0.82
Uccheddu (2019) [71]	9	7.00 ± 0.45	77.80	ASD	Reading-to-dogs program, with no touching permitted, 1 × 30-min sessions per week over 10 weeks.	Random assignment to one of two conditions, following matching according to demographics and symptom severity: reading to dog; reading without a dog.	WISC-IV Working Memory Index	No significant difference in WM.

*Note*. ^a^ = dissertation or thesis; ^b^ = diagnoses included but not limited to ASD, ADHD, Down’s syndrome, and global development delay; ^c^ = children attending alternative school due to previous suspension, expulsion, or behavioural difficulties; ^d^ = diagnoses included but not limited to ASD, ADHD, disruptive mood regulation disorder, and intellectual disability; ^e^ = work by Schroeder (2015) included both child and adult sample with results analysed separately. ANT = Attention Network Test; ANT-C = Attention Network Task, Child Version; BRIEF = Behaviour Rating Inventory of Executive Function; BRIEF-2 = Behaviour Rating Inventory of Executive Function, Second Edition; BRIEF-A = Behaviour Rating Inventory of Executive Function, Adult Version; BRIEF-P = Behaviour Rating Inventory of Executive Function, Preschool Version; fMRI = Functional Magnetic Resonance Imaging; WCST = Wisconsin Card Sorting Task; WISC-IV = Wechsler Intelligence Scale for Children (fourth edition). * *p* < 0.05. ** *p* < 0.01. *** *p* < 0.001.

**Table 5:** Quality assessment for *n* = 13 studies examining the impact of an animal-assisted service.

**Critical Appraisal Tool for Assessment of Risk of Bias for Randomised Controlled Trials**	**Study**									
	**Aviv** **(2021) [74]**	**Borgi** **(2016) [75]**		**Dimolareva** **(2020) [76]**	**Park** **(2019) [68]**		**Pendry** **(2021) [67]**	**Tepper** **(2021) [79]**		**Uccheddu** **(2019) [71]**
1. Was true randomisation used for assignment of participants to treatment groups?	Unclear. Authors only state randomisation occurred.	Unclear. Authors only state simple randomisation occurred.		Unclear. Authors only state randomisation occurred.	Yes		Unclear. Authors only state randomisation occurred.	Unclear. Authors only state randomisation occurred.		Unclear. Authors only state randomisation occurred.
2. Was allocation to treatment groups concealed?	Unclear	Unclear		Unclear	Yes		Unclear	Yes		Unclear
3. Were treatment groups similar at the baseline?	Yes	Despite randomisation, groups differed at baseline.		Yes	Despite randomisation, groups differed at baseline.		Yes	Yes		Yes
4. Were participants blind to treatment assignment?	No	No		No	No		Blinded to ratio of HAI	No		No
5. Were those delivering the treatment blind to treatment assignment?	No	No		No	No		No	No		No
6. Were treatment groups treated identically other than the intervention of interest?	Yes	Yes		Yes	Yes		Yes	Yes		Yes
7. Were outcome assessors blind to treatment assignment?	Yes	Yes		Unclear	Unclear		Unclear	Yes		No
8. Were outcomes measured in the same way for treatment groups?	Yes	Yes		Yes	Yes		Yes	Yes		Yes
9. Were outcomes measured in a reliable way?	Yes	Yes		Yes	Yes		Yes	Yes		Yes
10. Was follow up complete, and if not, were differences between groups in terms of their follow-up adequately described and analysed?	Yes	Yes		Yes	Yes		Yes	Yes		Yes
11. Were participants analysed in the groups to which they were randomized?	No ITT stated	No ITT stated		No ITT stated	No ITT stated		Yes	No ITT stated		No ITT stated
12. Was appropriate statistical analysis used?	Yes	Yes		Yes	Yes		Yes	Yes		Yes
13. Was the trial design appropriate and any deviations from the standard RCT design (individual randomization, parallel groups) accounted for in the conduct and analysis of the trial?	Yes	Yes		Yes	Yes		Yes	Yes		Yes
**Checklist for Quasi-Experimental Studies (Nonrandomized Experimental Studies)**	**Study**									
	**Gilboa** **(2020) [77]**		**Norwood** **(2021) [70]**			**Panczykowski** **(2021) [78]**			**Schroeder** **(2015) [69]**	
1. Is it clear in the study what is the ‘cause’ and what is the ‘effect’ (i.e., there is no confusion about which variable comes first)?	Yes		Yes			Yes			No. Participants had participated in horseback riding before.	
2. Were the participants included in any comparisons similar?	Participants acted as own control		Participants acted as own control			Participants acted as own control			No	
3. Were the participants included in any comparisons receiving similar treatment/care other than the exposure or intervention of interest?	Not applicable		Not applicable			Not applicable			Unclear	
4. Was there a control group?	No		*n* = 9 participants had data collected 6-weeks prior to the program, allowing them to act as a control			No			Yes	
5. Were there multiple measurements of the outcome both before and after the intervention/exposure?	Yes		Yes			Yes			Yes	
6. Was follow-up complete, and if not, were differences between groups in terms of their follow-up adequately described and analysed?	Yes		Yes			Yes			Yes	
7. Were the outcomes of participants included in any comparisons measured in the same way?	Not applicable		Not applicable			Yes			Yes	
8. Were outcomes measured in a reliable way?	Yes		Yes			Yes			Yes	
9. Was appropriate statistical analysis used?	Yes		Yes			Yes			Yes	
**Checklist for Case Reports**	**Study**									
	**Koenigseder** **(2016) [72]**					**Naste** **(2018) [73]**				
1. Were patient’s demographic characteristics clearly described?	Yes					Yes				
2. Was the patient’s history clearly described and presented as a timeline?	No					Yes				
3. Was the current clinical condition of the patient on presentation clearly described?	Yes					Yes				
4. Were diagnostic tests or assessment methods and the results clearly described?	Yes					Yes				
5. Was the intervention(s) or treatment procedure(s) clearly described?	Yes					No				
6. Was the postintervention clinical condition clearly described?	Yes					Yes				
7. Were adverse events (harms) or unanticipated events identified and described?	Not applicable					Not applicable				
8. Does the case report provide takeaway lessons?	Yes					Yes				

*Note*. ITT = Intention-to-treat analysis.

## 4. Discussion

The purpose of this review was to explore whether interactions with animals can improve executive functions. A systematic review of peer-reviewed and grey literature yielded 23 studies addressing three broad research questions: (1) examining the association between the human–pet relationship and EFs, (2) testing whether EF task performance is improved in the presence of an animal, and (3) examining whether EFs improved following involvement in an animal-assisted service. Due to these different research questions as well as heterogeneity between research design, participants, outcome measures, and quality, a critical comparison of the literature was difficult. However, some trends did emerge.

First, three of the studies recruited older (60+ years) adults, with all studies suggesting that interacting with an animal can improve EFs across multiple domains, such as sustained attention and overall executive control [39,54,68]. In Branson et al.’s study, the directionality of the relationship between caring for a pet and EFs could not be determined; it is possible that caring for a pet improves EF skills, but it is also probable that older adults with better EFs are more likely to keep a pet [39]. Demeter’s study provided some further evidence that caring for a pet can help retain EF skills in older adults, with significant differences found between stroke survivors living with a pet versus stroke survivors without a pet, but this study was limited by a small sample size and convenience sampling [54]. The strongest evidence for the benefits of caring for a pet for EFs comes from Park et al. [68], in which older Korean adults who raised pet crickets over an eight-week period demonstrated improved EFs compared to participants in the control condition. Taken together, these three studies provide evidence supporting the benefits of the human–pet relationship on EFs for older adults. Due to the paucity of this research, however, further studies are clearly needed.

There was no research exploring the benefits of caring for a pet for adults aged 18–59 years, although two studies in this age group explored whether the direct presence of an animal could improve performance on WM tasks [60,64], and two additional studies explored the benefits of animal-assisted services [67,69]. Across these research questions, results were mixed. Nagasawa et al. found that interacting with a cat activated the prefrontal cortex, the brain area implicated in EFs, and postulated that this would generalise to improved EF skills [62]. In comparison, Thayer and Stevens [64] found no significant differences in WM skills when completing the task in the presence of an animal, while Gee et al. suggested that WM performance was poorest when participants were required to maintain contact with a dog [60]. For the long-term intervention studies, only Pendry and colleagues found significant improvements in EFs across time [67]. In addition to reflecting differences across study duration and time spent with an animal, this may also reflect that Pendry et al. recruited at-risk adult participants, including those with learning disabilities or mental health conditions, who may have benefitted more from EF training [67].

Two studies exploring the benefits of caring for a pet for children did not find any significant differences between pet caregivers and non–pet caregivers [38,55], unlike the research with older adults. One possible explanation for this difference is that adults are more likely to be responsible for pet care, with activities such as remembering to provide the correct amount of food for a pet possibly providing an opportunity to practise EF skills [38,39]. In comparison, young children are less likely to be the primary caregiver for a pet, while some research has suggested that adolescents become less involved with their pets as they get older [80,81]. While caring for a pet did not appear to improve EFs in children, some of the reviewed research suggests that the simple presence of an animal can improve task performance [59,61,63]. It is possible that the presence of an animal may reduce children’s feelings of stress and anxiety and provide a nonjudgemental source of support, thereby improving task performance, with this explanation arising from similar research exploring the benefits of reading-to-dog programs [41].

For research examining improvements to children’s EFs across time, several studies suggested that horseback riding improved EFs, including inhibition, WM, monitoring, and planning skills [70,74,77,78]. This supports previous research that found that horseback riding and equine-assisted therapy can improve self-regulatory behaviours, with a previous review suggesting improvements in hyperactivity, irritability, and task engagement in autistic children and adolescents [82]. The research on therapeutic programs incorporating a dog was less consistent; in one study, participants with a poorer baseline demonstrated significant improvements in EFs [79], while two studies found no significant differences [71,76]. Interestingly, most of the studies that reported improvements in EFs were longer-term programs, taking place over more than seven weeks [70,73,74,75,77,78], while a six-week program [72] and a four-week program [76] reported mixed and no significant findings, respectively.

Different research designs and outcome measures make it difficult to determine whom these interventions most benefit. It is also noteworthy that two studies, which measured EFs using parent reports and reports completed by horseback riding instructors, found that parents reported no significant improvements in their child’s EFs [69,78]. This may reflect that some EF skills do not transfer to other contexts, such as the home, or reflect bias within the instrument. In addition, the longer-term benefits of animal-assisted services remain unknown, with few of the reviewed studies conducting follow-up. A review by Diamond and Lee [21] suggested that the impacts of EF interventions diminish over time once the intervention is ceased, and it is possible that interacting with an animal may have no long-term benefits on the development of cognitive skills.

Overall, the studies exploring the potential benefits of human–animal interactions on executive functions was of moderate to high quality. We highlight that future research should address potential bias within measures and explore longer-term changes in EFs, while the broader human–animal interaction field would benefit from the inclusion of larger sample sizes, the use of randomised controlled trials, and appropriate randomisation and control conditions [83]. Unfortunately, we were unable to provide effect sizes for several papers in this review because the effect size had not been explicitly reported or the original paper did not present the appropriate statistics to compute an effect size. This has previously been highlighted as a concern in other human–animal interaction reviews [41,84], as statistical significance alone is not enough to determine the efficacy of using human–animal interactions to improve health, well-being, and functioning.

There are several limitations inherent in the current review. For example, the conceptualisation of EFs was limited to three core cognitive constructs: working memory, inhibition, and cognitive flexibility. While this three-factor model of EFs is prevalent within the literature [6], some researchers have suggested that EFs encompass additional constructs or domains [85]. In the present study, the inclusion of ‘executive function’ as a broad search team as well as our large initial search sample likely captured all papers exploring the relationship between human–animal interactions and EFs, but it is possible that some papers using a different terminology were missed. In the present study, we also only reviewed quantitative literature, thereby possibly missing research examining related constructs such as on-/off-task behaviour and regulatory behaviours [86]. Future reviews may therefore benefit from expanding the search terminology and exploring qualitative and observational research.

## 5. Conclusions

As executive functions are linked to academic and workplace success, adaptive functioning, and quality of life, it is important to continue exploring ways to improve these skills. Overall, the effects of interacting with animals on executive functions cannot be clearly determined from the current literature; however, there are some promising trends. In particular, it appeared that interacting with animals, encompassing caring for a pet and animal-assisted services, may benefit older adults. Additionally, there was some evidence to support the efficacy of horseback riding programs, particularly for at-risk children and adolescents. Higher-quality studies exploring the benefits of the human–pet relationship and the benefits of involvement in animal-assisted service are recommended, with a focus on incorporating randomised controlled trials or longitudinal study designs.

## Figures and Tables

**Table 1:** Study characteristics, methodology, and key findings for *n* = 4 studies examining the benefits of the human-animal relationship.

First Author (Year)	Participants				Pet Characteristics		Methodology	Outcome Measure	Outcomes
	*N*	Age in Years (*M ± SD*)	Gender (% Male)	Diagnosis or Risk	% Pet Caregivers	Pet Type			
Branson (2016) [39]	88	75.00 ± 9.00	34.10	Physical or mobility limitations	55.00	Dogs, cats, fish, birds, rodents	Cross-sectional design, comparing pet caregivers to non–pet caregivers.	CLOX 1	Significantly higher EF score for participants with a pet *, Cohen’s *d* = 0.44 (small effect).
Demeter (2020) ^a^ [54]	52	68.60 ± 7.66	69.20	Stroke	48.10	Dogs, cats, fish, birds	Cross-sectional design, comparing pet caregivers to non–pet caregivers.	CTT; Kettle Test	Significantly faster performance time on sustained attention task for participants with a pet *. No effect sizes reported by original author; effect size subsequently calculated by authors of this review as *r* = −0.31 (medium). No significant difference in divided attention or sequencing task.
Purewal (2019) ^a,b^ [55]	13,557 pet caregivers during gestatation	<17–>40 maternal age at delivery	0	Varied ^c^	Varied across time	Dogs, cats, fish, birds, rabbits, rodents, horses	Longitudinal design, with pet caregiving status collected at various time points.	TEA-Ch Opposite Worlds; Stop-signal task; Digit recall task; Counting span task	No significant association between caring for a pet and stronger cognitive outcomes across time.
Tepper (2022) [38]	207	9.38 ± 2.15	52.20	Varied ^d^	72.00	Dogs, cats, fish, birds, rabbits, rodents	Cross-sectional survey, comparing pet caregivers to non–pet caregivers.	CHEXI	No significant relationship between caring for a pet and WM or inhibition ability.

*Note*. ^a^ = dissertation or thesis; ^b^ = study by Purewal [55] reports on participants recruited from longitudinal ALSPAC birth cohort study; ^c^ = broader ALSPAC research reports diagnoses including but not limited to ASD and ADHD [56,57]; ^d^ = in Tepper et al.’s [38] study, 11.10% (*n* = 23) participants had a disability, with diagnoses including but not limited to ASD, ADHD, and dyslexia; CLOX 1 = Executive Clock Drawing Task 1; CHEXI = Child Executive Functioning Inventory; CTT = Colour Trail Test; TEA-Ch = Test of Everyday Attention for Children. * *p* < 0.05.

**Table 2:** Study characteristics, methodology, and key findings for *n* = 6 studies examining the impact of the presence of an animal.

First Author (Year)	Participants				Human–Animal Interaction Characteristics	Methodology	Outcome Measure	Outcomes
	*N*	Age in Years (*M ± SD*)	Gender (% Male)	Diagnosis or Risk				
Becker (2014) [59] ^a^	38	11.70 ± 1.90	89.47	Neurodevelopmental and behavioural disorders	3 min of direct interaction; two dogs included in study.	Repeated measure design, with participants spending time with a real dog versus a stuffed toy dog control.	WISC-IV coding task, WRAML-2 picture memory task, and NEPSY-II Inhibition subtest	Significantly fewer errors on cognitive flexibility task * and fewer errors on incongruent inhibition task * when in presence of the real dog. No effect sizes provided by original author; effect sizes subsequently calculated by authors of this review as Cohen’s *d* = −0.54 (medium) for cognitive flexibility and *d* = 0.47 (medium) for inhibition.
Gee (2015) [60]	31	Not reported (18–23 years)	16.1	None	Total time spent with animal not reported; two dogs included in study.	Repeated measure design, with participants completing task in five counterbalanced conditions: touching a dog, dog present with no touching, touching a person, person present with no touching, and alone.	iPad^®^ iMimic Challenge	Significantly poorer WM score when touching dog *.
Oostendorp Godfrey (2020) ^a^ [61]	7	7.5 ± Not reported	57.10	Undisclosed learning disabilities	Total time spent with animal not reported; one dog included in study.	Repeated measure design with participants completing task when dog was present and absent.	TOMAL 2 digit/letter span	Mixed findings reported for individual WM profiles, but no *p*-values or effect sizes reported by original author. Subsequent analyses by authors of this review revealed no statistically significant group difference.
Hediger (2014) [63]	24	11.34 ± 0.95	54.20	None	15 min spent with animal; one dog included in study.	Repeated measure crossover design, with participants spending time with real dog versus robotic dog. Study took place over two consecutive weeks.	WISC-IV digit span (German version), CANDIT attention task, and PIR HEG	Significant improvement in WM when real dog was present in the second test session *, Cohen’s *d* = 0.51 (medium effect). Greater PIR HEG signal in presence of real dog.
Nagasawa (2020) [62]	29	21.17 ± 0.65	34.50	None	4 min spent with animal; one cat included in study.	Single-subject design, with participants interacting with a cat in four broad ways: touching the cat, playing with the cat, training the cat, and feeding the cat. This was compared with data collected pre–post interacting with the cat.	fNRIS	Significant change in prefrontal cortex activation when interacting with cat, regardless of interaction type **. No effect sizes provided.
Thayer (2022) ^b^ [64]	Ex^1^: 73 Ex^2^: 83	Ex^1^: 19.2 ± 1.40 Ex^2^: 19.9 ± 1.80	Ex^1^: 17.80 Ex^2^: 20.50	None	3 min spent with animal; one dog included in study.	Pseudo-randomly assigned to one of two conditions: interaction with a dog or control condition, in which participants circled every ‘e’ and ‘f’ on a page full of Latin text.	NCPC, Backwards digit span, and N-back	Ex^1^: No significant difference in WM. Ex^2^: No significant difference in WM.

*Note*. ^a^ = dissertation or thesis; ^b^ = publication by Thayer et al. [64] reports on two experiments (Ex^1^ and Ex^2^ = experiment one and experiment two), with the same outcome measures and research design, but different participants; CANDIT = Computer Assisted Neuropsychological Diagnostics and Therapy; fNIRS = Functional Near-Infrared Spectroscopy; NCPC = Necker Cube Pattern Control; NEPSY-II = A Developmental NeuroPsychological Assessment (second edition); PIR HEG = Passive Infrared Hemoencephalography; RCT = Randomised Controlled Trial; WISC-IV = Wechsler Intelligence Scale for Children (fourth edition); WRAML-2 = Wide Range Assessment of Memory and Learning (second edition). * *p* < 0.05. ** *p* < 0.01.

**Table 3:** Quality assessment for *n* = 6 studies examining the impact of the presence of an animal.

**Critical Appraisal Tool for Assessment of Risk of Bias for Randomised Controlled Trials**	**Study**			
	**Becker** **(2014) [59]**	**Gee** **(2015) [60]**	**Hediger** **(2014) [63]**	**Thayer** **(2022) [64]**
1. Was true randomisation used for assignment of participants to treatment groups?	Unclear. Authors only state that randomisation occurred.	Unclear. Authors only state that randomisation occurred.	Unclear. Authors only state that randomisation occurred.	Unclear. Authors only state that randomisation occurred.
2. Was allocation to treatment groups concealed?	Yes	Yes	Unclear	Yes
3. Were treatment groups similar at the baseline?	Yes	Yes	Yes	Yes
4. Were participants blind to treatment assignment?	No	No	Blinded to aim of study	No
5. Were those delivering the treatment blind to treatment assignment?	No	No	No	No
6. Were treatment groups treated identically other than the intervention of interest?	Yes	Yes	Yes	Yes
7. Were outcome assessors blind to treatment assignment?	No	No	Unclear	No
8. Were outcomes measured in the same way for treatment groups?	Yes	Yes	Yes	Yes
9. Were outcomes measured in a reliable way?	Yes	Yes, but not valid	Yes	Yes
10. Was follow-up complete, and if not, were differences between groups in terms of their follow-up adequately described and analysed?	Yes	Yes	Yes	Yes
11. Were participants analysed in the groups to which they were randomized?	No ITT stated	No ITT stated	No ITT stated	No ITT stated
12. Was appropriate statistical analysis used?	Yes	Yes	Yes	Yes
13. Was the trial design appropriate and were any deviations from the standard RCT design (individual randomization, parallel groups) accounted for in the conduct and analysis of the trial?	Yes	Yes	Yes	Yes
**Checklist for Quasi-Experimental Studies (Non-Randomized Experimental Studies)**	**Study**			
	**Nagasawa** **(2020) [62]**		**Oostendorp Godfrey** **(2020) [61]**	
1. Is it clear in the study what is the ‘cause’ and what is the ‘effect’ (i.e., there is no confusion about which variable comes first)?	Yes		Yes	
2. Were the participants included in any comparisons similar?	Participants acted as own control		Participants acted as own control	
3. Were the participants included in any comparisons receiving similar treatment/care other than the exposure or intervention of interest?	Not applicable		Not applicable	
4. Was there a control group?	No		No	
5. Were there multiple measurements of the outcome both before and after the intervention/exposure?	Yes		Yes	
6. Was follow up complete and if not, were differences between groups in terms of their follow up adequately described and analysed?	Yes		Yes	
7. Were the outcomes of participants included in any comparisons measured in the same way?	Not applicable		Not applicable	
8. Were outcomes measured in a reliable way?	Yes		Yes	
9. Was appropriate statistical analysis used?	Yes		Yes	

*Note*. ITT = Intention-to-treat Analysis.

## Data Availability

Not applicable.

## References

[1] Diamond A. (2013). Executive functions. Annu. Rev. Psychol..

[2] Diamond A., Gallagher A., Bulteau C., Cohen D., Michaud J.L. (2020). Executive functions. Handbook of Clinical Neurology.

[3] Josman N., Meyer S. (2019). Conceptualisation and use of executive functions in paediatrics: A scoping review of occupational therapy literature. Aust. Occup. Ther. J..

[4] Best J.R., Miller P.H. (2010). A developmental perspective on executive function. Child Dev..

[5] Diamond A., Bialystok E., Craik F.I.M. (2006). The early development of executive functions. Lifespan Cognition: Mechanisms of Change.

[6] Miyake A., Friedman N.P., Emerson M.J., Witzki A.H., Howerter A., Wager T.D. (2000). The unity and diversity of executive functions and their contributions to complex “frontal lobe” tasks: A latent variable analysis. Cogn. Psychol..

[7] Carlson S.M. (2005). Developmentally sensitive measures of executive function in preschool children. Dev. Neuropsychol..

[8] Gerstadt C.L., Hong Y.J., Diamond A. (1994). The relationship between cognition and action: Performance of children 3½–7 years old on a stroop-like day-night test. Cognition.

[9] Huizinga M., Dolan C.V., Van der Molen M.W. (2006). Age-related change in executive function: Developmental trends and a latent variable analysis. Neuropsychologia.

[10] Kochanska G., Aksan N. (1995). Mother-child mutually positive affect, the quality of child compliance to requests and prohibitions, and maternal control as correlates of early internalization. Child Dev..

[11] Ferguson H.J., Brunsdon V.E., Bradford E.E. (2021). The developmental trajectories of executive function from adolescence to old age. Sci. Rep..

[12] Phillips L.H., Henry J.D., Anderson V., Jacobs R., Anderson P.J. (2008). Adult aging and executive functioning. Executive Functions and the Frontal Lobes: A Lifespan Perspective.

[13] Davis J.C., A Marra C., Najafzadeh M., Liu-Ambrose T. (2010). The independent contribution of executive functions to health related quality of life in older women. BMC Geriatr..

[14] Otero T.M., Barker L.A., Naglieri J.A. (2014). Executive function treatment and intervention in schools. Appl. Neuropsychol. Child.

[15] Darby R.R., Dickerson B.C. (2017). Dementia, decision-making, and capacity. Harv. Rev. Psychiatry.

[16] McDermott L.M., Ebmeier K.P. (2009). A meta-analysis of depression severity and cognitive function. J. Affect. Disord..

[17] Snyder H.R. (2013). Major depressive disorder is associated with broad impairments on neuropsychological measures of executive function: A meta-analysis and review. Psychol. Bull..

[18] Aupperle R.L., Melrose A.J., Stein M.B., Paulus M.P. (2012). Executive function and PTSD: Disengaging from trauma. Neuropharmacology.

[19] Lawrence V., Houghton S., Douglas G., Durkin K., Whiting K., Tannock R. (2004). Executive function and ADHD: A comparison of children’s performance during neuropsychological testing and real-world activities. J. Atten. Disord..

[20] Hill E.L. (2004). Executive dysfunction in autism. Trends Cogn. Sci..

[21] Diamond A., Lee K. (2011). Interventions shown to aid executive function development in children 4 to 12 years old. Science.

[22] Alexander P., Berri A., Moran D., Reay D., Rounsevell M.D. (2020). The global environmental paw print of pet food. Glob. Environ. Change.

[23] Friesen L. (2010). Exploring animal-assisted programs with children in school and therapeutic contexts. Early Child. Educ. J..

[24] Yakimicki M.L., Edwards N.E., Richards E., Beck A.M. (2019). Animal-assisted intervention and dementia: A systematic review. Clin. Nurs. Res..

[25] Rusanen M., Selander T., Kärkkäinen V., Koivisto A. (2021). The positive effects of pet ownership on Alzheimer’s Disease. J. Alzheimer’s Dis..

[26] Nieforth L.O., Schwichtenberg A., O’Haire M.E. (2021). Animal-assisted interventions for autism spectrum disorder: A systematic review of the literature from 2016 to 2020. Rev. J. Autism Dev. Disord..

[27] O’Haire M.E. (2013). Animal-assisted intervention for autism spectrum disorder: A systematic literature review. J. Autism Dev. Disord..

[28] O’Haire M.E. (2017). Research on animal-assisted intervention and autism spectrum disorder, 2012–2015. Appl. Dev. Sci..

[29] Pérez-Gómez J., Amigo-Gamero H., Collado-Mateo D., Barrios-Fernandez S., Muñoz-Bermejo L., Garcia-Gordillo M., Carlos-Vivas J., Adsuar J.C. (2021). Equine-assisted activities and therapies in children with attention-deficit/hyperactivity disorder: A systematic review. J. Psychiatr. Ment. Health Nurs..

[30] Busch C., Tucha L., Talarovicova A., Fuermaier A.B.M., Lewis-Evans B., Tucha O. (2016). Animal-assisted interventions for children with attention deficit/hyperactivity disorder: A theoretical review and consideration of future research directions. Psychol. Rep..

[31] Kaminski M., Pellino T., Wish J. (2002). Play and pets: The physical and emotional impact of child-life and pet therapy on hospitalized children. Child. Health Care.

[32] Wu A.S., Niedra R., Pendergast L., McCrindle B.W. (2002). Acceptability and impact of pet visitation on a pediatric cardiology inpatient unit. J. Pediatr. Nurs..

[33] Barker S.B., Knisely J.S., McCain N.L., Best A. (2005). Measuring stress and immune response in healthcare professionals following interaction with a therapy dog: A pilot study. Psychol. Rep..

[34] Staats S., Wallace H., Anderson T. (2008). Reasons for companion animal guardianship (pet ownership) from two populations. Soc. Anim..

[35] Gilbey A., Tani K. (2015). Companion animals and loneliness: A systematic review of quantitative studies. Anthrozoös.

[36] Friedmann E., Gee N.R., Simonsick E.M., Studenski S., Resnick B., Barr E., Kitner-Triolo M., Hackney A. (2020). Pet ownership patterns and successful aging outcomes in community dwelling older adults. Front. Vet. Sci..

[37] le Roux M., Boyd L. (2017). ‘When he’s up there he’s just happy and content’: Parents’ perceptions of therapeutic horseback riding. Afr. J. Disabil..

[38] Tepper D.L., Howell T.J., Bennett P.C. (2022). Executive functions and household chores: Does engagement in chores predict children’s cognition?. Aust. Occup. Ther. J..

[39] Branson S., Boss L., Cron S., Kang D.-H. (2016). Examining differences between homebound older adult pet owners and non-pet owners in depression, systemic inflammation, and executive function. Anthrozoös.

[40] Brelsford V.L., Meints K., Gee N.R., Pfeffer K. (2017). Animal-assisted interventions in the classroom—A systematic review. Int. J. Environ. Res. Public Health.

[41] Hall S.S., Gee N.R., Mills D.S. (2016). Children reading to dogs: A systematic review of the literature. PLoS ONE.

[42] Blankenship T.L., Slough M.A., Calkins S.D., Deater-Deckard K., Kim-Spoon J., Bell M.A. (2019). Attention and executive functioning in infancy: Links to childhood executive function and reading achievement. Dev. Sci..

[43] Voss S.E., Bullock R.A. (2004). Executive function: The core feature of dementia?. Dement. Geriatr. Cogn. Disord..

[44] Willcutt E.G., Doyle A.E., Nigg J.T., Faraone S.V., Pennington B.F. (2005). Validity of the executive function theory of attention-deficit/hyperactivity disorder: A meta-analytic review. Biol. Psychiatry.

[45] Moher D., Liberati A., Tetzlaff J., Altman D.G., PRISMA Group (2009). Preferred reporting items for systematic reviews and meta-analyses: The PRISMA statement. Ann. Intern. Med..

[46] Folstein M.F., Folstein S.E., McHugh P.R. (1975). “Mini-mental state”: A practical method for grading the cognitive state of patients for the clinician. J. Psychiatr. Res..

[47] Barker T.H., Stone J.C., Sears K., Klugar M., Tufanaru C., Leonardi-Bee J., Aromataris E., Munn Z. (2023). The revised JBI critical appraisal tool for the assessment of risk of bias for randomized controlled trials. JBI Evid. Synth..

[48] Tufanaru C., Munn Z., Aromataris E., Campbell J., Hopp L., Aromataris E., Munn Z. (2020). Systematic reviews of effectiveness. JBI Manual for Evidence Synthesis.

[49] Moola S., Munn Z., Tufanaru C., Aromataris E., Sears K., Sfetic R., Currie M., Lisy K., Qureshi R., Mattis P., Aromataris E., Munn Z. (2020). Chapter 7: Systematic reviews of etiology and risk. JBI Manual for Evidence Synthesis.

[50] Chen F.-T., Etnier J.L., Chan K.-H., Chiu P.-K., Hung T.-M., Chang Y.-K. (2020). Effects of exercise training interventions on executive function in older adults: A systematic review and meta-analysis. Sport. Med..

[51] Lund J.I., Toombs E., Radford A., Boles K., Mushquash C. (2020). Adverse childhood experiences and executive function difficulties in children: A systematic review. Child Abus. Negl..

[52] Lakens D. (2013). Calculating and reporting effect sizes to facilitate cumulative science: A practical primer for t-tests and ANOVAs. Front. Psychol..

[53] Pallant J., Unwin A. (2013). SPSS Survival Manual: A Step by Step Guide to Data Analysis.

[54] Demeter N. (2020). Attachment orientation towards a pet in stroke survivors: Association with cognitive function, participation and quality of life. Ph.D. Thesis.

[55] Purewal R. (2019). Companion Animals and Child Development: Existing Knowledge and Analysis of the Avon Longitudinal Study of Parents and Children Cohort. Ph.D. Thesis.

[56] Martin J., Hamshere M.L., Stergiakouli E., O’donovan M.C., Thapar A. (2014). Genetic risk for attention-deficit/hyperactivity disorder contributes to neurodevelopmental traits in the general population. Biol. Psychiatry.

[57] Williams E., Thomas K., Sidebotham H., Emond A. (2008). Prevalence and characteristics of autistic spectrum disorders in the ALSPAC cohort. Dev. Med. Child Neurol..

[58] Ma L.L., Wang Y.Y., Yang Z.H., Huang D., Weng H., Zeng X.T. (2020). Methodological quality (risk of bias) assessment tools for primary and secondary medical studies: What are they and which is better?. Mil. Med. Res..

[59] Becker J.L. (2014). Presence of a dog on executive functioning and stress in children with emotional disorders. Ph.D. Thesis.

[60] Gee N.R., Friedmann E., Coglitore V., Fisk A., Stendahl M. (2015). Does physical contact with a dog or person affect performance of a working memory task?. Anthrozoös.

[61] Oostendorp Godfrey J. (2020). Dogs, Working Memory and Educational Achievement: Barking up the Wrong Tree or an Effective Mechanism for Facilitating Cognitive Acuity?. Ph.D. Thesis.

[62] Nagasawa T., Ohta M., Uchiyama H. (2020). Effects of the characteristic temperament of cats on the emotions and hemodynamic responses of humans. PLoS ONE.

[63] Hediger K., Turner D.C. (2014). Can dogs increase children’s attention and concentration performance? A randomised controlled trial. Hum. Anim. Interact. Bull..

[64] Thayer E.R., Stevens J.R. (2022). Effects of human-animal interactions on affect and cognition. Hum. Anim. Interact. Bull..

[65] Skowronek J.S., Leichtman M.D., Pillemer D.B. (2008). Long-term episodic memory in children with Attention-Deficit/Hyperactivity Disorder. Learn. Disabil. Res. Pract..

[66] Evans G.W., Schamberg M.A. (2009). Childhood poverty, chronic stress, and adult working memory. Proc. Natl. Acad. Sci. USA.

[67] Pendry P., Carr A.M., Vandagriff J.L., Gee N.R. (2021). Incorporating human-animal interaction into academic stress management programs: Effects on typical and at-risk college students’ executive function. AERA Open.

[68] Park J.-Y., Ko H.-J., Kim A.-S., Moon H.-N., Choi H.-I., Kim J.-H., Chang Y., Kim S.-H. (2019). Effects of pet insects on cognitive function among the elderly: An fMRI study. J. Clin. Med..

[69] Schroeder V.M. (2015). Giddy-up your cognitive processes: The influence of horseback riding as a physical activity on executive functioning. Ph.D. Thesis.

[70] Norwood M.F., Lakhani A., Maujean A., Downes M., Fullagar S., Barber B.L., Kendall E. (2021). The horse as a therapist: Effects of an equine program without “therapy” on the attention and behavior of youth disengaged from traditional school. J. Altern. Complement. Med..

[71] Uccheddu S., Albertini M., Pierantoni L., Fantino S., Pirrone F. (2019). The impacts of a Reading-to-Dog Programme on attending and reading of nine children with Autism Spectrum Disorders. Animals.

[72] Koenigseder M. (2016). Outcomes in language and social skills as seen in children with autism and developmental disabilities participating in equine assisted activities. Bachelor’s Thesis.

[73] Naste T.M., Price M., Karol J., Martin L., Murphy K., Miguel J., Spinazzola J. (2018). Equine facilitated therapy for complex trauma (EFT-CT). J. Child Adolesc. Trauma.

[74] Aviv T.-L.M., YKatz J., Berant E. (2021). The contribution of therapeutic horseback riding to the improvement of executive functions and self-esteem among children with ADHD. J. Atten. Disord..

[75] Borgi M., Loliva D., Cerino S., Chiarotti F., Venerosi A., Bramini M., Nonnis E., Marcelli M., Vinti C., De Santis C. (2016). Effectiveness of a standardized equine-assisted therapy program for children with autism spectrum disorder. J. Autism Dev. Disord..

[76] Dimolareva M. (2020). Animal-Assisted Interventions in Special Needs Schools: What Works?. Ph.D. Thesis.

[77] Gilboa Y., Helmer A. (2020). Self-management intervention for attention and executive functions using equine-assisted occupational therapy among children aged 6–14 diagnosed with attention deficit/hyperactivity disorder. J. Altern. Complement. Med..

[78] Panczykowski H., Murphy L., Adams K., Bralley M., Millner L. (2021). The impact of an interactive vaulting equine program on executive function and group participation of children with disabilities: A mixed methods pilot study. Altern. Complement. Ther..

[79] Tepper D.L., Connell C.G., Landry O., Bennett P.C. (2021). Dogs in schools: Can spending time with dogs improve executive functioning in a naturalistic sample of young children?. Anthrozoös.

[80] Mathers M., Canterford L., Olds T., Waters E., Wake M. (2010). Pet ownership and adolescent health: Cross-sectional population study. J. Paediatr. Child Health.

[81] Muldoon J.C., Williams J.M., Lawrence A. (2015). ‘Mum cleaned it and I just played with it’: Children’s perceptions of their roles and responsibilities in the care of family pets. Childhood.

[82] Tan V.X.-L., Simmonds J.G. (2019). Equine-assisted interventions for psychosocial functioning in children and adolescents with autism spectrum disorder: A literature review. Rev. J. Autism Dev. Disord..

[83] Rodriguez K.E., Herzog H., Gee N.R. (2021). Variability in human-animal interaction research. Front. Vet. Sci..

[84] Reilly K.M., Adesope O.O., Erdman P. (2020). The effects of dogs on learning: A meta-analysis. Anthrozoös.

[85] Baggetta P., Alexander P.A. (2016). Conceptualization and operationalization of executive function. Mind Brain Educ..

[86] Moffett L., Morrison F.J. (2020). Off-task behavior in kindergarten: Relations to executive function and academic achievement. J. Educ. Psychol..

